# Outcome of intracranial arterial stenting of symptomatic atherosclerotic disease: A single center experience from Saudi Arabia

**DOI:** 10.17712/nsj.2016.4.20160199

**Published:** 2016-10

**Authors:** Youssef Al Said, Khalil Kurdi, Saleh S. Baeesa, Ahmed Najjar, Mohammed Almekhlafi, Ahmed Hassan

**Affiliations:** *From the Divisions of Neurosurgery (Baeesa), Divisions of Neurology (Almekhlafi), Faculty of Medicine, King Abdulaziz University, and from the Department of Neurosciences (Al Said, Najjar, Hassan) Department of Radiology (Kurdi), King Faisal Specialist Hospital and Research Center, Jeddah, Kingdom of Saudi Arabia*

## Abstract

**Objectives:**

To present our local experience with intracranial angioplasty and stenting used for the treatment of symptomatic intracranial stenosis to assess its safety, efficacy, and outcome.

**Methods:**

This is a retrospective review of all the patients with symptomatic intracranial atherosclerotic disease who underwent endovascular treatment in King Faisal Specialist Hospital and research center, Jeddah, Kingdom of Saudi Arabia from January 2003 to December 2014. Clinical, procedural, and outcome variables were gathered.

**Results:**

We identified 22 patients who were referred for stenting of symptomatic intracranial atherosclerotic stenosis. In all but 3, the stents were deployed successfully (86% procedural success rate). The procedure was carried out under conscious sedation in 32%. Excellent flow was restored immediately in all successfully-stented cases. Post procedural strokes occurred in 4 patients (17.4%). One non-neurological death was identified in a patient who suffered a major post procedural stroke (4.3%).

**Conclusion:**

Intracranial atherosclerotic disease is not uncommon in our population. Angioplasty and stenting might be a valid option for the treatment of patients with recurrent symptoms despite optimal medical treatment.

The natural history of intracranial atherosclerotic disease has a dynamic nature of progression and regression. The risk of stroke of all causes in patients with intracranial stenosis can be as high as 10-24% per year, despite optimal medical therapy.^1^-^4^ Recurrent ischemic events typically occur within a short period after failure of standard therapy.^3^ This high risk of recurrent stroke on medical therapy prompted the search for alternative treatment methods. Cerebral percutaneous transluminal angioplasty (PTA) with or without stent placement has been performed in patients with symptomatic intracranial atherosclerosis who fail standard medical therapy. This procedure was a promising treatment option to prevent recurrence of ischemic stroke. However, the enthusiasm for PTA has been halted by the result of the Stenting versus Aggressive Medical Therapy for Intracranial Arterial Stenosis “SAMMPRIS” trial.^5^ Stent-assisted angioplasty carries a spectrum of procedure-related cerebrovascular complications. These complications include intracerebral hemorrhage, target–lesion thrombosis, perforator stroke, embolic stroke, Transient Ischemic Attacks (TIA) and vessel dissection or perforation. The objective of our study is to evaluate the cerebrovascular complications of angioplasty with stent placement for symptomatic intracranial stenosis.

## Methods

Following Institutional review board approval, we retrospectively reviewed the medical records of all patients who underwent endovascular treatment for symptomatic atherosclerotic intracranial stenosis between January 2003 and December 2014 performed at King Faisal Specialist Hospital and research center, Jeddah, Kingdom of Saudi Arabia. All patients with symptomatic intracranial vascular stenosis more than 50% were included. We excluded cases with other potential cause of stroke or TIA such as cardioembolic events or vasospasm. Patients’ demographic, timing of cerebral ischemic event, stroke risk factors, and location and severity of intracranial atherosclerosis were identified. Hypertension, defined as receiving blood pressure medication for blood pressure more than 140/90, diabetes, cardiac disease, and hyperlipidaemia were all noted. Current smoking was defined as ongoing or within 6 months of quitting. Antiplatelet or anticoagulants use was noted prior to and after the procedure.

Brain and cerebrovascular imaging including CT, CT angiography (CTA), MRI, MR angiography (MRA), and conventional cerebral digital subtraction angiography (DSA) were reviewed for all included patients. The intracranial stenosis was defined as the presence of 50-99% stenosis on CTA, MRA, or angiography. The stenosis was considered to be symptomatic when the brain imaging showed a relevant regional infarct ipsilateral and distal to the vascular territory of the stenotic vessel or if the patient’s symptoms are attributed to that territory.

The occurrence of adverse events was evaluated during the procedure, immediately post-procedure, before discharge, and at 30-days. Adverse events were divided into minor strokes with new non-disabling neurological deficits, major stroke with persistent disabling deficits and death. The success of the stenting procedure was defined as no or less than 50% residual stenosis in the original disease segment. Procedure success was defined as stent success with no stroke or death before discharge. In addition, all patients received maximum medical treatment consisting of dual antiplatelet therapy plus lipid lowering agents. These are in addition to blood pressure control and glycemic control in patients with hypertension and diabetes. Six months then annual follow up radiological imaging including CT, CTA, MRI, MRA or angiography of the treated lesion were reviewed. Target lesion related stroke at 12 months from the procedure was also determined when available. Descriptive statistical tests were used as appropriate to summarize the variables as means or proportions.

## Results

Out of 22 patients included, the procedures were successfully performed in 19 patients. The failed procedures in 3 patients were due to technical difficulty caused by vascular tortuosity or vasospasm. There were 14 males and 5 females. The mean age at procedure was 58.3 years, with a range between 39 to 79 years. All patients were Saudis except one. All patients had at least one risk factor for atherosclerosis. Hypertension and diabetes were most common as the hypertension found in 74% patients and the diabetes 68% (**Table 1**).

**Figure 1:**
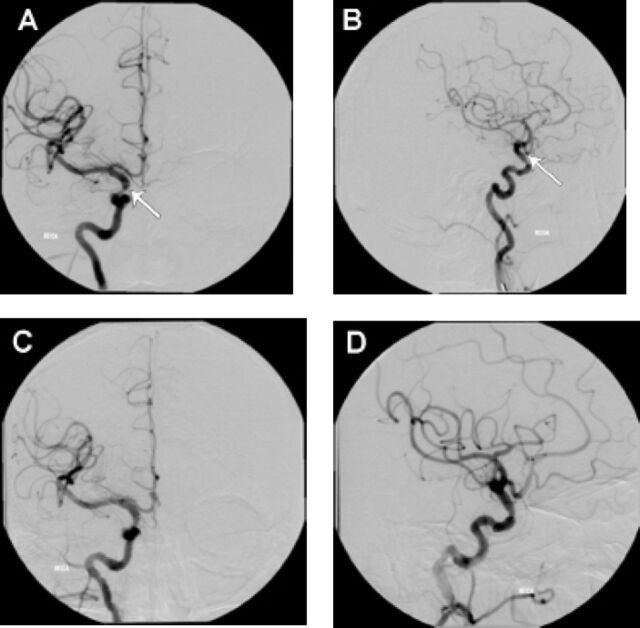
Case illustration of anterior circulation stenting, a 54-year-old male (patient # 20) presented with left hemiparesis due to right ICA 80% stenosis at the supraclinoid segment; arrows shows **a)** the segment of severe stenosis at the supraclinoid segment of the internal carotid artery, **b)** resolution of the stenosis after the stent placement, **c-d)** The patient underwent percutaneous angioplasty and stent placement with ferrous stent with good resolution of the stenotic part.

**Table 1:** Clinical, procedural and outcome details of the studied patients.

Age (sex) / clinical history	Vessel affected (% of stenosis based on DSA)	Endovascular treatment (anesthesia)	Type of stent (length/mm)	Outcome/complication (follow up)
1. 67 (F) / Slurred speech and right arm weakness.	Left M1-MCA (80%)	Angioplasty and stenting, GA	Taxus (2x10 mm)	Excellent (16 months) No new infarction or stent stenosis.
2. 61 (M) / Lower limbs paresis with facial weakness.	Mid-Basilar artery (85%)	Angioplasty and stenting, GA	Taxus (3x12 mm)	Died (6 months) from post-procedural pontine infarction.
3. 56 (M) / Right side hemiparesis.	Mid-basilar artery (80%)	Angioplasty and stenting, GA	Cardio stent (3.5x18 mm)	Excellent (4 years) No new infarction or stent stenosis.
4. 76 (M) / Right hemiplegia and recurrent TIA’s	Mid-basilar artery (60%)	Angioplasty and stenting, GA	Endeavor cardiac stent (2.5x8 mm)	Excellent (16 months) No new infarction or stent stenosis.
5. 65 (M) / Previous stroke of VBA origin	Mid-basilar artery (60%)	Angioplasty and stenting, GA	Failed (tortuous VA and severe spasm)	NA
6. 45 (M) / Recurrent right hemiparesis	Mid-basilar artery (60%) Right petro-cavernous-ICA (80%)	Angioplasty and stenting, GA	Wing span stents: 3.5x15 mm (for mid-basilar) 4 x 15 mm (for ICA)	Died (one month) Post-procedural acute right Pons infarction
7. 56 (M) / Recurrent left side TIA’s	Left supraclinoid ICA (75%).	Angioplasty and stenting, LA	Cypher Select Drug Eluting stent (2.5x13 mm)	Excellent (3 years) No new infarction or stent stenosis.
8. 79 (F) / Recurrent attacks of dizziness diplopia and ptosis	Right vertebral basilar junction (60%)	Angioplasty and Stenting, GA	Taxux Liberte (2.75x16 mm)	Excellent (4 years) No new infarction or stent stenosis.
9. 54 (M) / Recurrent attacks of incoordination and unsteady gait	Left proximal IC vertebral artery (80%)	Angioplasty and stenting, LA	Taxus (2.5x16 mm)	Good (3 years) No restenosis. Left MCA infarction due to severe right ICA stenosis (90%). No intervention due to renal failure.
10. 39 (M) / left sided weakness	Right cavernous-ICA (80%)	Angioplasty and stenting, LA	Precise Nitinol stent Taxus stent (2.5x10 mm)	Good (2 years) No new infarction or stent stenosis.
11. 63 (M) / Recurrent dysarthria and unsteady gait	Right Vertebrobasilar junction artery (80%)	Angioplasty and stenting, LA	Taxus (2.5x16 mm)	Excellent (2 years) No new infarction or stent stenosis.
12. 55 (F) / Recurrent TIAs	Left Vertebrobasilar junction artery (80%)	Angioplasty and stenting, LA	Taxus (3 x 20 mm)	Poor (12 months) Post-procedural right sided hemiplegia Lt pontine infarction, patent stent.
13. 47 (M) / Recurrent right side hemiparesis	Proximal vertebral artery (80%)	Angioplasty and stenting, GA	Driver RX cardiac stent 3.5x24 mm	Excellent (2 years) No new infarction or stent stenosis.
14. 42 (F) / Right upper limb weakness.	left ophthalmic-ICA (85%)	Angioplasty and stenting, GA	Taxus (3x20 mm)	Excellent (18 months) No new infarction or stent stenosis.
15. 70 (M) / Recurrent left side weakness	Right cavernous-ICA and proximal basilar artery stenosis (70% each)	Angioplasty and stenting, GA For right ICA	Driver RX cardio stent (3.5x18 mm)	Excellent (2 years) No new infarction or stent stenosis.
16. 67 (M) / Recurrent TIA’s	Right Vertebrobasilar junction artery (> 90%)	Angioplasty and stenting, GA	Taxus (3.5x12 mm)	Excellent (16 months) No new infarction or stent stenosis.
17. 55 (F) / Right hemiparesis (on warfarin)	Left M1- MCA (>80%)	Angioplasty and stenting, LA	Failed, (MCA spasm)	Poor (1 year) Developed stroke on the same site.
18. 43 (M) / Sudden LOC for 8 hrs	Basilar artery (>90%)	Angioplasty (+tPA) and stenting GA	Solitaire (2.5x16 mm)	Poor (3 months) Died due to massive SAH.
19. 68 (M) / Decrease LOC, walking difficulty.	Right Vertebrobasilar junction (80%)	Angioplasty and stenting, GA	Failed due to vascular tortuosity and spasm	NA
20. 54 (M) / Left hemiparesis	Right Supraclinoid ICA (80 %)	Angioplasty and stenting, LA	FERROUS (3x20 mm)	Excellent (2 years) No new infarction or stent stenosis.
21. 65 (M) / Recurrent dysarthria and mild quadriparesis	Distal basilar artery (90%)	Angioplasty and stenting, GA	Abbott Xience Prime drug eluting (2.5x16 mm)	Excellent (14 months) No new infarction or stent stenosis.
22. 69 (M) / Recurrent TIA’s	Proximal basilar artery (90%)	Angioplasty and stenting, GA	Taxus Liberte (3x20 mm)	Excellent (1 year) No new infarction or stent stenosis.

F - Female, M - male, TIA - Transient Ischemic Attacks, MCA - middle cerebral artery, ICA - Internal Carotid Artery, GA - general anesthesia, LA - local anesthesia, NA - Not applicable, LOC - level of consciousness, tPA - tissue plasminogen activator

**Figure 2:**
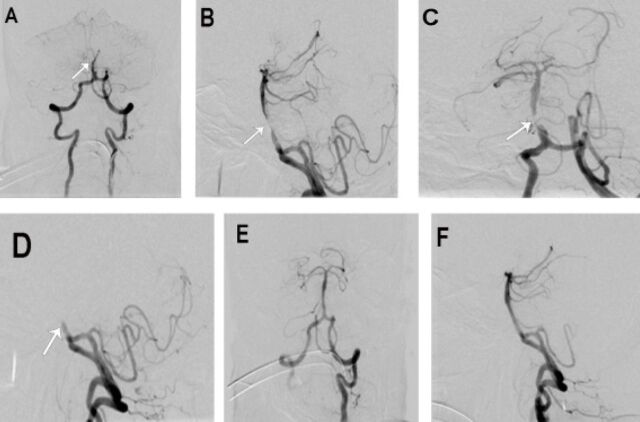
Case illustration of posterior circulation stenting, a 69-year-old male (patient # 22) presented with recurrent vertebrobasilar insufficiency due to near occlusion of mid basilar artery, **a-d)** He underwent percutaneous angioplasty; arrows shows the segment of near occlusion at the mid basilar artery. and **e-f)** Showed good patency of the basilar artery after stent placement with Taxus Liberte.

### Pre-procedure antiplatelets/anticoagulants

All patients were on antiplatelet therapy, except one who was on warfarin. One patient was non-compliant to the antiplatelet medications and was on inconsistent intake of Aspirin. All patients had intracranial stenosis with symptoms referable to that stenosis territory. The events were major stroke in 5 patients (22.7%) and 3 other patients had minor events in the form of recurrent TIA and minor strokes. One patient had recurrent posterior circulation TIA with no evidence of stroke in the posterior circulation, however this patient had 2 stenotic lesions one involving left Internal Carotid Artery (ICA) extra cranially with secondary documented multiple strokes distal to this territory, and another lesion involving the left vertebral artery intracranially. Both lesions were stented at the same time with no complications and with successful recanalization.

### Interventional procedure

All 22 procedures were carried out by a single neurointerventionist and summarized in **Table 2**. Seven procedures were carried out under conscious sedation (32%). Stent success defined as less than 50 % stenosis involving a segment no longer than the original lesion was achieved in 17 of the 19 patients; the procedure was terminated in 3 patients due to vascular tortuosity or spasm. Angiographic runs taken after the procedure were done for all patients. Stent placement success rate was 100 % with good flow and recanalization in all interventions. Four patients (17.4%) developed postoperative complications early in the form of major stroke. One of the patients who suffered a stroke died after one month due to respiratory complications. The rest of patients were discharged home with no stroke representing 77.2% of treated patients. Excellent outcomes were documented in 11 patients with a minimum follow up of 6 months. All patients with anterior circulation intracranial stenting had no post procedural complications and all 4 patients with complications had posterior circulation stenting.

## Discussion

Intracranial arterial stenosis is a worldwide disease, responsible for 6-10% of ischemic strokes in Whites, 6-29% of ischemic strokes in Blacks, 11% of ischemic strokes in Hispanics, and 22-26% of ischemic strokes in Asians.^6^ Once thought to be uncommon, it is now known that intracranial atherosclerotic disease is almost as common as extracranial carotid atherosclerotic disease in some populations.^7^ Patients with symptomatic intracranial severe stenosis have a risk of up to 14% of suffering future intracranial ischemic insults in the subsequent 2 years despite maximum medical therapy.^8^ The annual risk for subsequent stroke may exceed 20% in high-risk groups. Groups with the highest risk of recurrent stroke are those with high-grade (> or = 70%) stenosis, those with recent symptom onset, women, those with vertebrobasilar disease with history of diabetes mellitus, and patients who fail anti-thrombotic therapy.^6^ The mechanism of stroke in these patients may be related to thromboembolism owing to unstable plaque, hemodynamic factors leading to flow reduction beyond the stenosis, or synergistic effects of the two.^6^

For patients who present within days to weeks after their qualifying event, percutaneous transluminal angioplasty and stenting (PTAS) was introduced as a potential mean for secondary stroke prevention.^9^ Preliminary studies showed that angioplasty and stenting reduced the risk of stroke in patients with severe stenosis of intracranial arteries.^6^,^10^,^11^ However, the endovascular approach was evaluated in a phase 3 randomized controlled trial which was stopped after 451 patients were randomized. The SAMMPRIS trial randomized patients with severe stenosis affecting major intracranial artery to undergo aggressive medical management alone versus aggressive medical management plus angioplasty and stenting (with Wingspan stent). The trial outcome of 30-day stroke or death was significantly higher in the endovascular arm (14.7%) compared to the medical arm (5.8%) leading to the early stoppage of the trial.^6^

While some practitioners strongly advocate the use of angioplasty alone, i.e., without a stent, most favor the use of angioplasty with stenting. The addition of stenting to the angioplasty procedure results in a substantially greater overall improvement in the final luminal diameter. While many “technically successful” PTA procedures leave behind 50% residual stenosis or more, after a PTAS procedure the residual Stenosis is typically 10% or less.^9^

Three types of stents have been used in the intracranial circulation. 1. Balloon-expandable bare-metal stents or balloon mounted coronary stents: these stents were not designed for the intracranial use. When deployed, these balloon-expandable stents often distort the regional anatomy and sometimes exert significant trauma particularly within tortuous vascular segments. Their use was associated with high rates of morbidity and mortality reaching 30% due to technical factors mentioned. Recently, an intracranial balloon-expandable stents were tested in a randomized controlled trial (VISSIT trial) in patients with symptomatic intracranial stenosis. The trial was stopped prematurely due to higher rates of 30-day and one-year stroke or TIA in the stenting arm.^12^ 2. Drug-eluting balloon expandable stents: these were a major breakthrough in preventing the risk of ISR but they are associated with subacute and late stent thrombosis.^6^,^10^,^13^ Also, prolonged use of Aspirin and Plavix is required with drug eluting stents. This could increase the risk of intracerebral hemorrhage. 3. Self expanding Stents: Based on the European Asian Wingspan Study and the approval of Wingspan Stent by the Food and Drug Administration (FDA), these stents are the currently indicated stents for intracranial use to treat patients with symptomatic intracranial atherosclerotic disease who failed medical treatment. It is currently in the United States under the FDA Humanitarian Device Exemption for patients with severe symptomatic intracranial atherosclerotic stenosis who failed maximum medical therapy. In our series we had more experience with drug eluting stents with good and acceptable results. However, these stents were not allowed in the SAMMPRIS trial. This was one of the points raised by the critics of this trial.^14^ Complication rates are highly variable and we recommend that this procedure be used in highly specialized centers with the capability to manage pre, intra- and post procedure issues.

This report has limitations. This is retrospective case series which may introduce biases related to documentations and missing information. In addition, these are cases done in a single center which may limit the generalizability of the results. However, we also believe that reporting our findings have merits. There are no reports describing the volume or outcome of intracranial stenting from local centers over a long period demonstrating the real-life experience of performing these specialized procedures. There remains a number of areas for future research regarding intracranial stenting. The optimal time and stent choice for patients with symptomatic intracranial stenosis are yet to be defined. Any differences between the anterior versus posterior circulation in natural history and prognosis could be instructive toward defining high risk groups for future events despite maximum medical therapy.

In Conclusion, the endovascular treatment for symptomatic intracranial stenosis remains in the development phase in light of the recent evidence. However, in the small subset of patients who are refractory to maximum medical treatment, stenting is an option to be considered with appropriate patient selection and good experience of the interventionist.
